# Bipolar Dislocation of the Arm (Shoulder And Elbow): About One Case in an African Teaching Hospital

**DOI:** 10.2174/1874325001812010147

**Published:** 2018-03-30

**Authors:** DA Songahir Christophe, Korsaga Alexandre, Tinto Sayouba, Sawadogo Mamoudou, Kafando Hamado, Ouedraogo Anatole Jean Innocent, Zeida Charles, Keita Namori, Tall Mohamed

**Affiliations:** 1 Yalgado OUEDRAOGO University Hospital, 03 BP 7022 Ouagadougou 03, Burkina Faso; 2 Ouahigouya Regional University Hospital Center Ouahigouya, BP 36, Burkina Faso; 3 Blaise COMPAORE University Hospital 11 BP 104 Ouagadougou CMS 11, Burkina Faso

**Keywords:** Fracture, Dislocation, Bipolar, Shoulder, Elbow, Swinging arm

## Abstract

We report an exceptional clinical case of an ipsilateral dislocation fracture of the shoulder and right elbow, realizing a “swinging arm”. Following a violent road accident, patient S.I, a 43-year-old left-handed sports educator, presented with an antero-medial shoulder dislocation fracture and a posterolateral ipsilateral elbow fracture-dislocation. The reduction in urgency, followed by the orthopedic compression by Mayo Clinic and functional rehabilitation, allowed obtaining a good result after seven months. The ipsilateral bipolar dislocation of the shoulder and elbow is an exceptional lesional entity. Its adequate care in emergency makes it possible to obtain good anatomical and functional results.

## 1 INTRODUCTION

 Bipolar dislocations of the thoracic limb are rare lesions resulting from violent trauma [1-5]. It is an emergency the treatment of which consists of a reduction and stabilization of the lesions, followed by functional re-education of the traumatized limb. The authors report an exceptional clinical case of an ipsilateral dislocation fracture of the shoulder and right elbow, realizing a swinging arm.

## 2 OBSERVATION

 The 43-year-old sports educator, S.I. left-handed, suffered a road traffic accident on February 27, 2016. He was a passenger on a transit bus that would have rolled several times. This accident caused a staged closed trauma of the right thoracic limb. The exact mechanism of injury could not be specified. He had sharp pains in the right shoulder and right elbow, with absolute functional impotence of the right thoracic limb. He was rushed by ambulance to the teaching hospital Yalgado OUEDRAOGO, trauma department about three hours after his trauma. The physical examination returned to Desault's attitude with deformity and swelling of the right shoulder and elbow. The vasculo-nervous examination was normal. The X-rays of the shoulder and the elbow, AP and lateral views, revealed anterior medial dislocation fracture of the shoulder Fig. (**1**) and a posterolateral dislocation fracture of the elbow Fig. (**2**).

The anteromedial fracture-dislocation of the right shoulder involved a right glenohumeral anteromedial dislocation and a fracture of the right major tubercle. The posterolateral dislocation fracture of the elbow consisted of a posterolateral elbow dislocation and a complex fracture of the right coronoid process. Treatment consisted of emergency orthopedic reduction, under general anesthesia, of the shoulder and elbow injuries, followed by immobilization elbow to the body by Mayo-Clinic Fig. (**3**). 

Radiographic control images showed satisfactory reductions in both the shoulder Fig. (**4**) and the elbow Fig. (**5**), with a non-anatomical reduction of the coronoid process fracture. The immobilization was maintained for six weeks and then the patient received 25 functional rehabilitation sessions of the right shoulder and elbow.

At seven months' follow-up, the shoulder was painless with normal mobility Fig. (**6**). At the elbow, the patient reported moderate pain during efforts. Prono-supination and flexion were almost normal, but the patient had a 7 ° elbow extension deficit Figs. (**7** and **8**).

Radiographic control images at the follow-up of seven months showed a consolidation of bone lesions in both the shoulder and the elbow Fig. (**9**).

## 3 DISCUSSION

 The bipolar dislocation fracture of the shoulder and elbow producing an ipsilateral “swinging arm” is exceptional. We have not found a similar case in the literature. The few rare cases of bipolar dislocation of the thoracic limb reported in the literature relate to the forearm (elbow and wrist) [2]. These lesions are due to violent trauma and require emergency management, by reducing and restraining lesions [1, 2, 4, 5]. Open reintegration of the fracture of the coronoid process followed by additional compression by plaster splint, would have improved anatomical and above all functional results.

## CONCLUSION

 The ipsilateral bipolar dislocation of the shoulder and elbow is an exceptional lesional entity whose mechanism of occurrence remains complex in the course of a violent trauma. Its emergency management makes it possible to obtain good anatomical and functional results.

## Figures and Tables

**Fig. (1):**
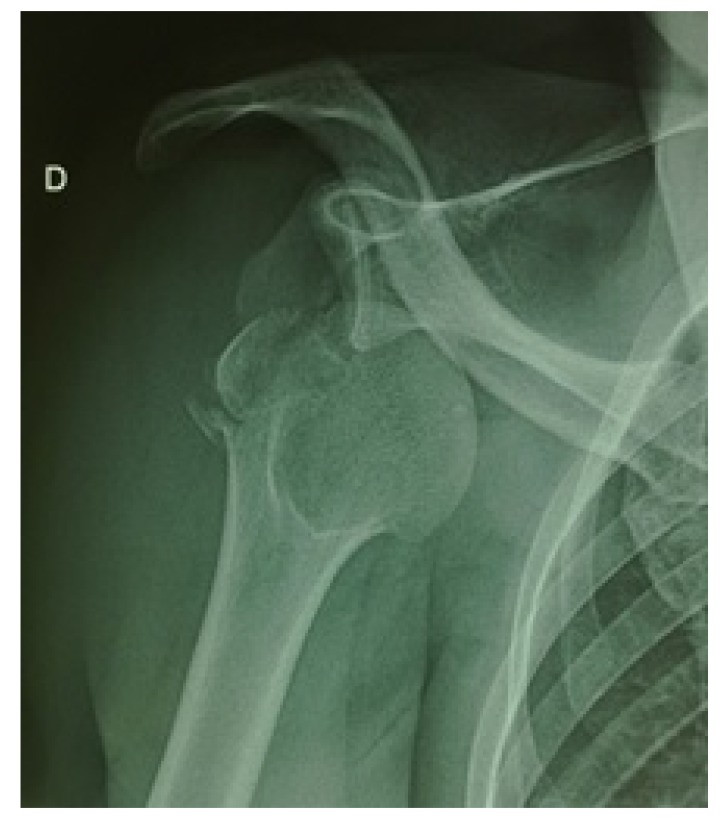
Anteromedial fracture dislocation of right shoulder

**Fig. (2):**
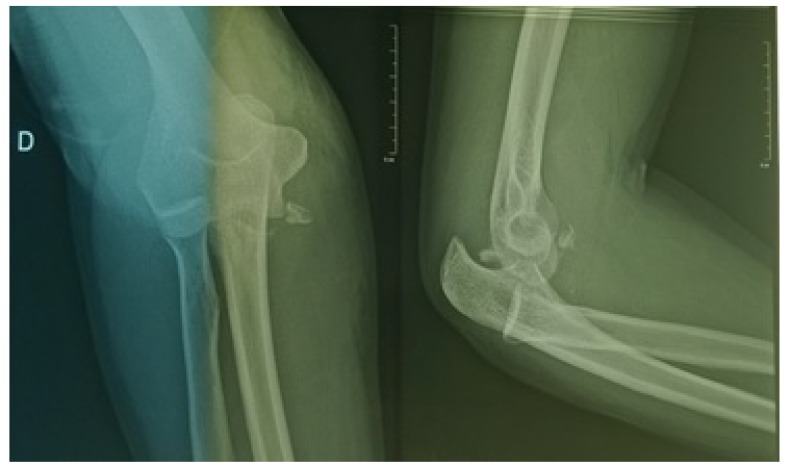
Posterolateral fracture dislocation of the right elbow.

**Fig. (3):**
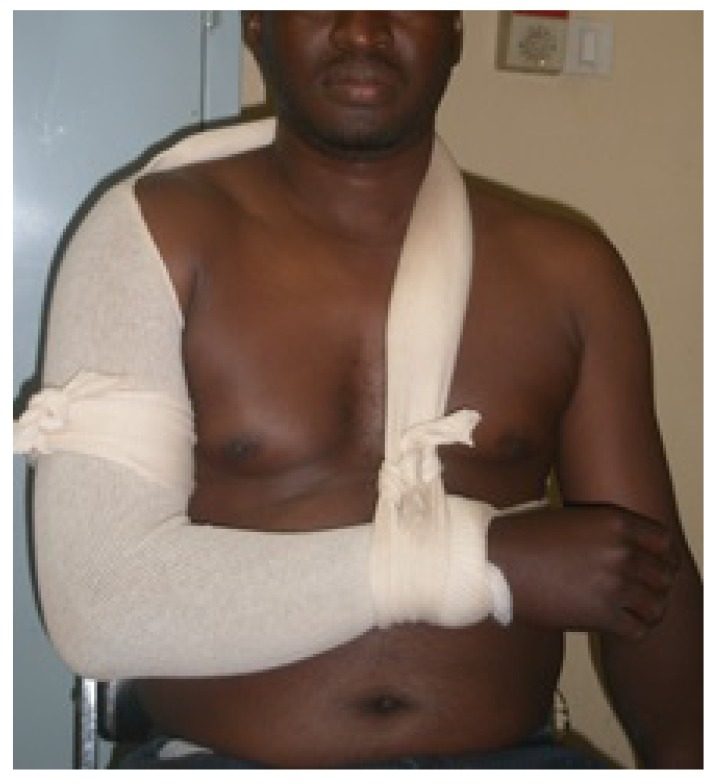
Immobilizing by Mayo-Clinic.

**Fig. (4):**
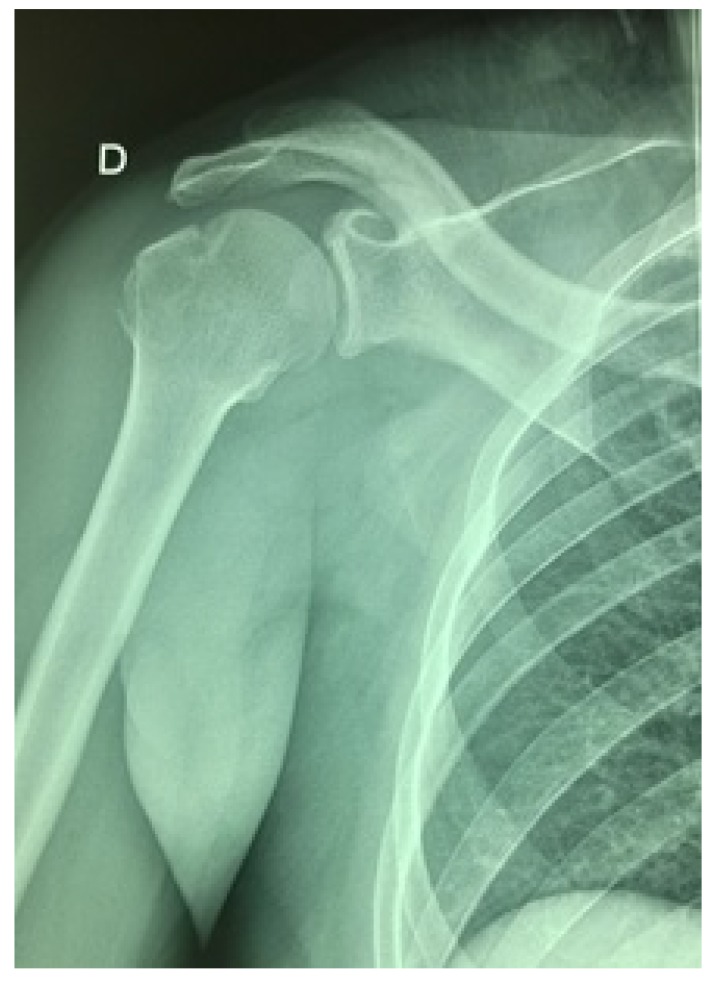
Post-reductional AP X-ray control of the fracture-dislocation of the shoulder.

**Fig. (5):**
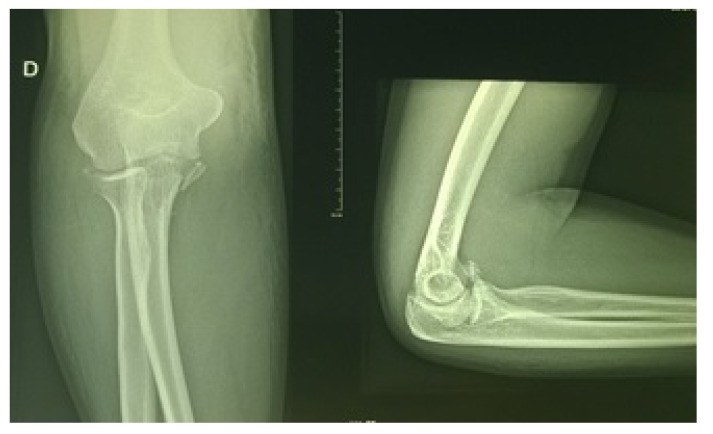
Post-reductional X-ray of the elbow fracture-luxation.

**Fig. (6):**
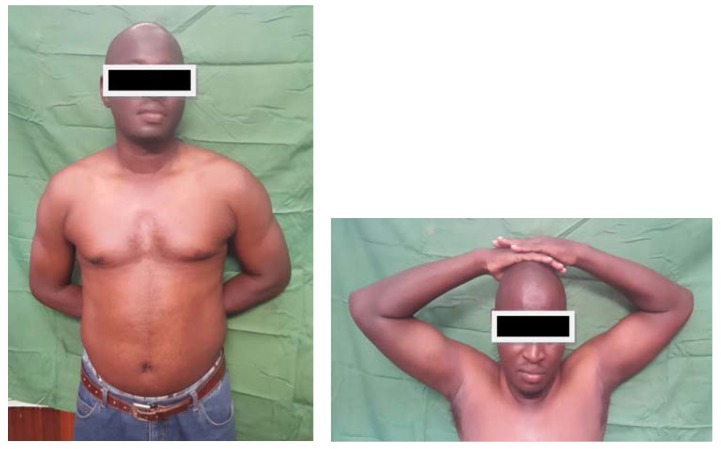
Satisfactory functional result of the shoulder in the 7th month.

**Fig. (7):**
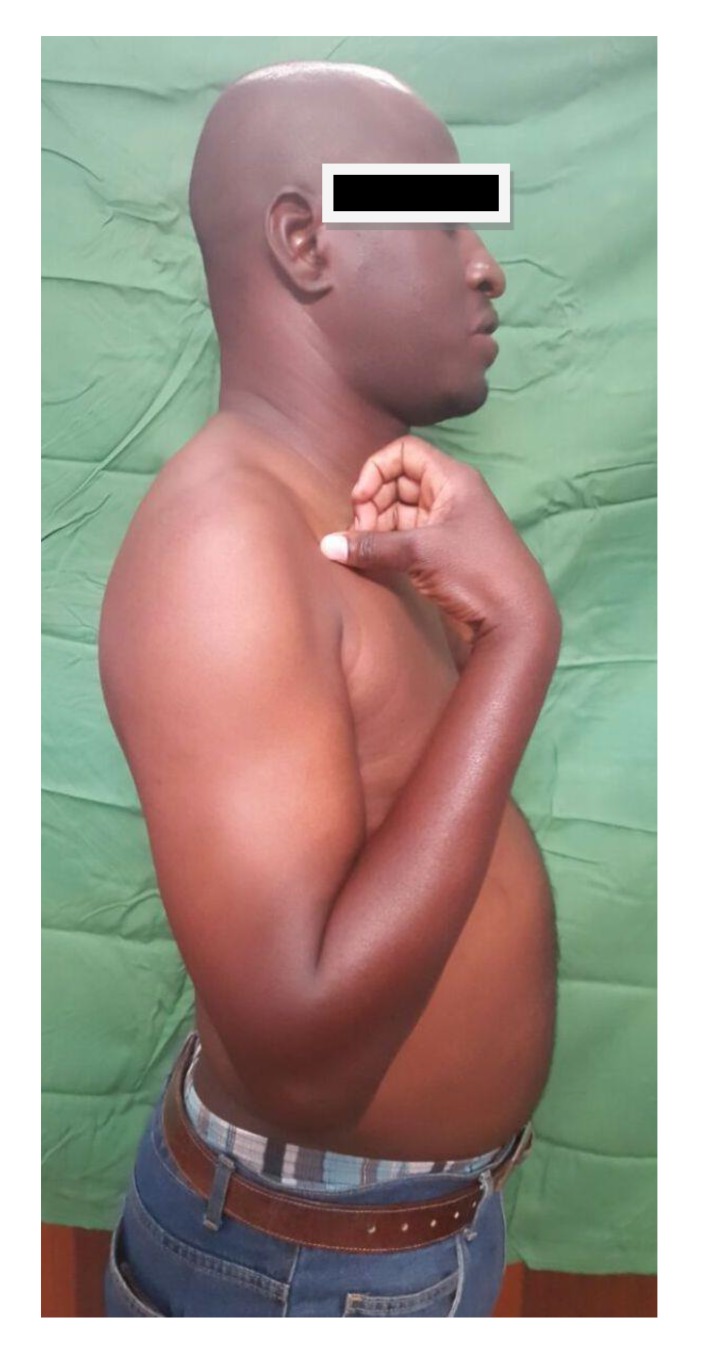
Very good elbow flexion at seven months follow-up.

**Fig. (8):**
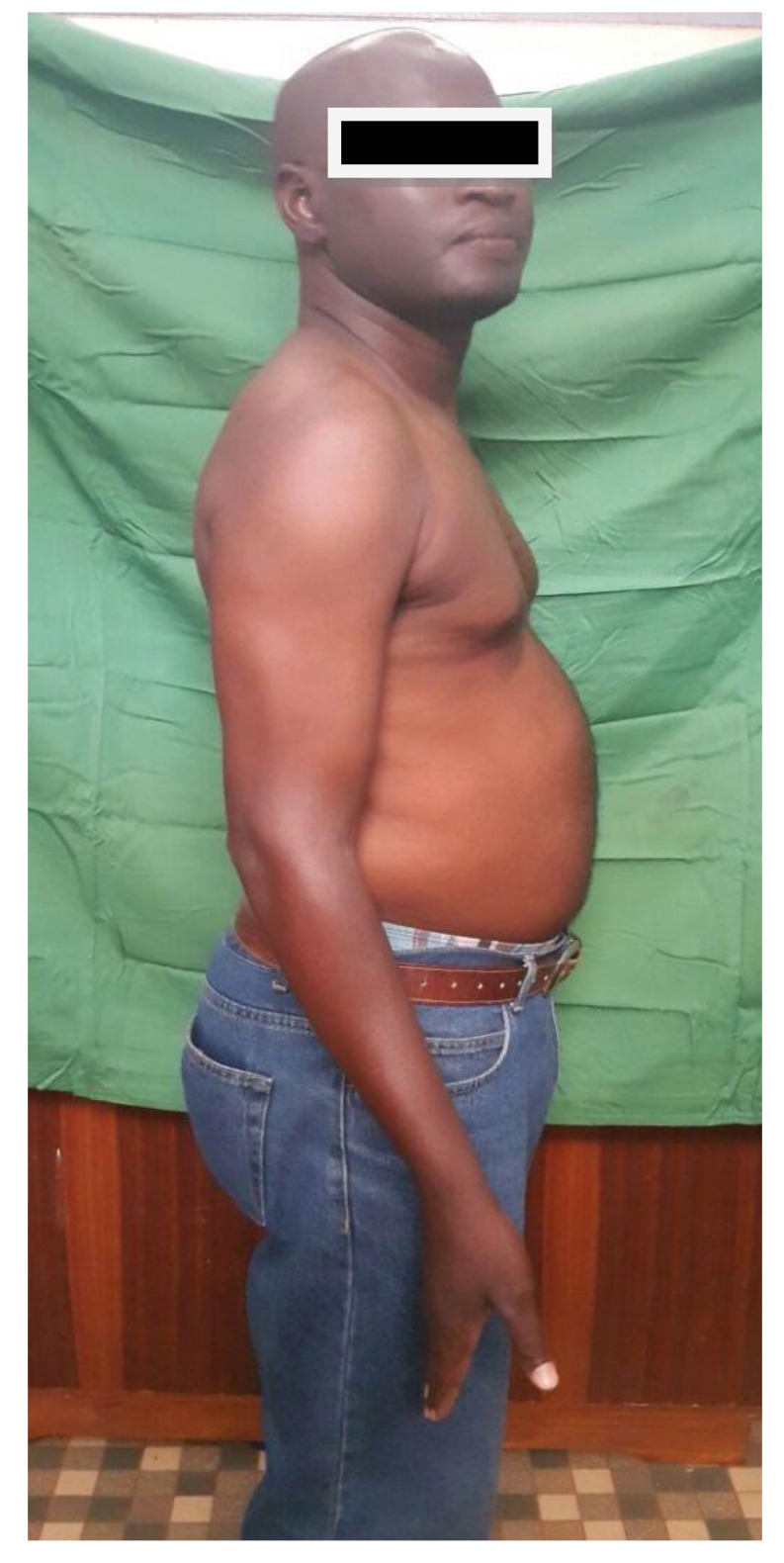
Slight deficit in elbow exten-sion at seven months' follow-up.

**Fig. (9):**
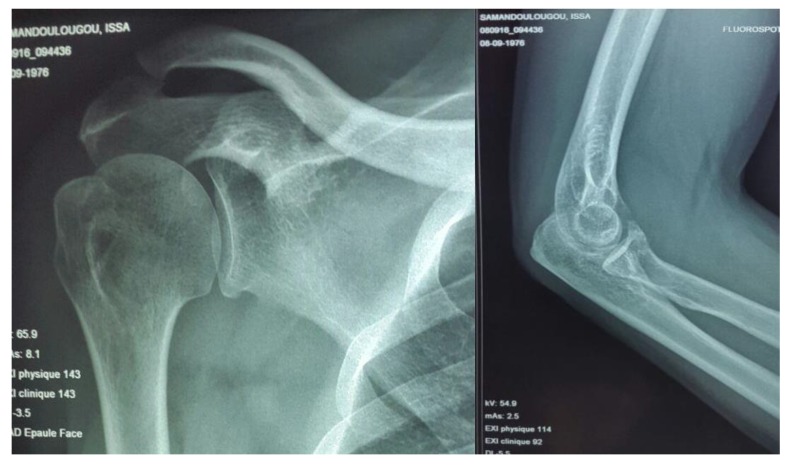
Bone consolidation of the elbow and shoulder at seven months follow-up.
